# Early Outcomes, Learning Curve and Cost Analysis of Totally Robotic Sleeve Gastrectomy

**DOI:** 10.1007/s11695-025-08427-x

**Published:** 2025-12-19

**Authors:** Antonio Vitiello, Giovanna Berardi, Maria Spagnuolo, Daniele Bruno, Marcello Persico, Vincenzo Pilone

**Affiliations:** 1 University of Naples Federico II, Naples, Italy; 2 Federico II University Hospital, Naples, Italy

**Keywords:** Robotic sleeve gastrectomy, Learning curve, CUSUM analysis, Perioperative outcomes, Cost analysis

## Abstract

**Background:**

Robotic sleeve gastrectomy (RSG) is gaining adoption in metabolic bariatric surgery, yet evidence on its perioperative safety, efficiency, and costs during the early learning phase remains limited.

**Objectives:**

To evaluate outcomes, learning curve dynamics, and procedural costs for totally robotic sleeve gastrectomy.

**Setting:**

Academic center.

**Methods:**

A retrospective analysis was conducted of the first 53 consecutive RSGs (April 2023–July 2025) performed by a single surgeon using a standardized fully robotic technique. Perioperative variables, 30-day morbidity, and cost data were collected. Learning curves for docking time, console time, and total operative time were assessed using Cumulative Sum (CUSUM) analysis.

**Results:**

Mean age and BMI were 38.1 ± 12.6 years and 41.6 ± 6.3 kg/m^2^, respectively; 71.7% were female. Overall complication rate was 7.5% (3 bleedings, 1 leak) with no mortality or reinterventions. ICU admission and readmission each occurred in 1.9% of cases. Mean docking time was 17.8 ± 9.4 min, console time 51.5 ± 17.0 min, and total operative time 69.3 ± 18.1 min. The mean length of stay was 3.6 ± 1.1 days (range 2–10). CUSUM identified proficiency points at case 18 for docking time (− 51.1%), case 23 for console time (− 18.6%), and case 22 for total operative time (− 21.5%). Median length of stay was 3 days. Mean direct instrument/stapling cost per RSG was €4,535.

**Conclusions:**

Totally RSG can be introduced with safe outcomes even during the learning phase. Proficiency for operative efficiency was reached after 20 cases.

**Graphical Abstract:**

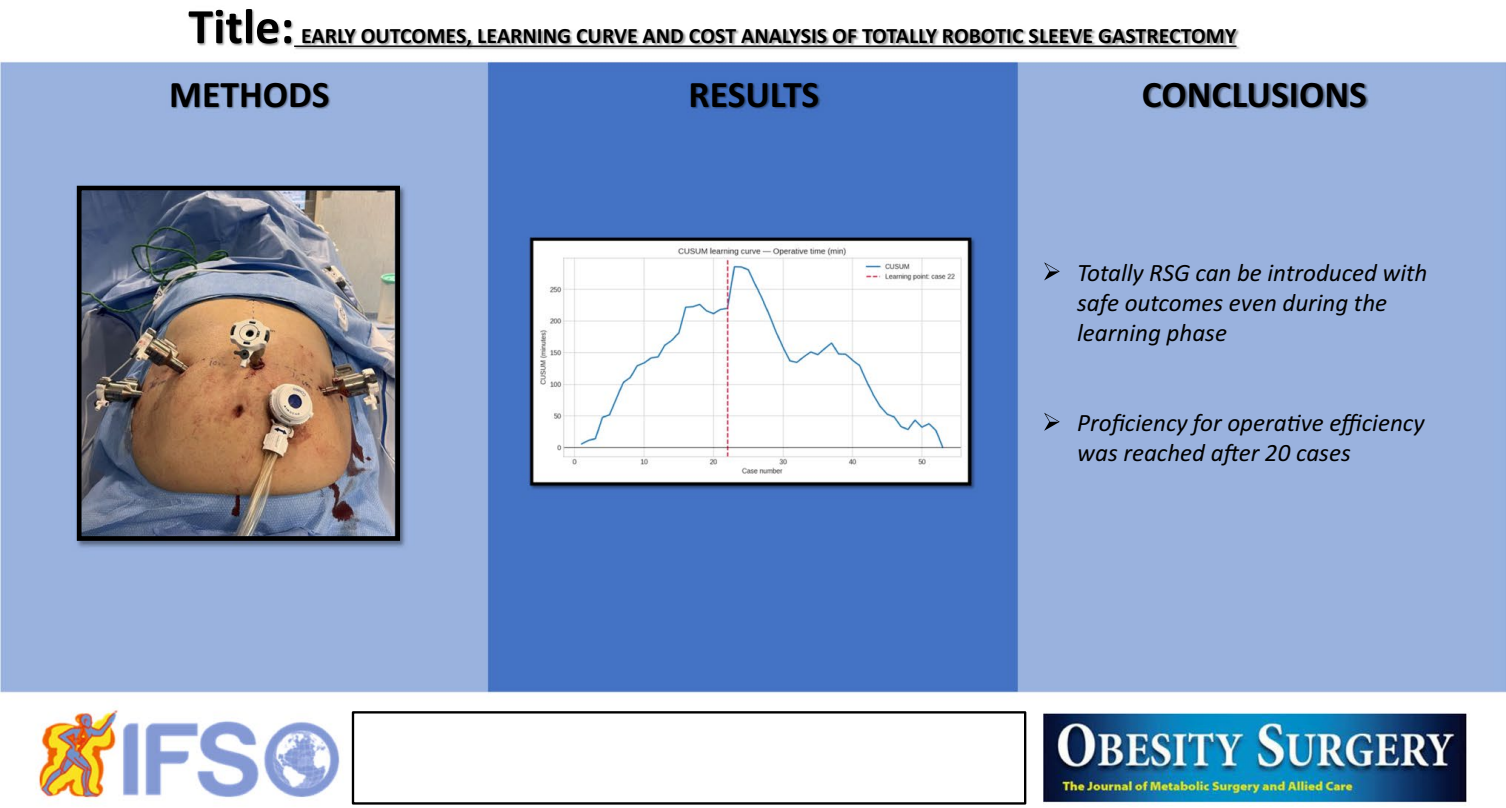

## Highlights

Overall complication rate was 7.5% (3 bleedings, 1 leak), with no mortality or reinterventions. Mean docking, console, and total operative times were 17.8, 51.5, and 69.3 minutes, respectively. CUSUM analysis showed proficiency reached after ~20 cases for operative efficiency.

## Introduction

Laparoscopic sleeve gastrectomy (LSG) continues to be the most frequently performed metabolic surgical procedure worldwide [1], representing more than half of all bariatric interventions in numerous countries [2]. Given its established laparoscopic feasibility, bariatric surgeons remain strongly anchored to the laparoscopic domain of minimally invasive surgery. According to recent IFSO report [3], in the United States robotic approaches account for 23% of all primary metabolic procedures, whereas in many European countries—including Italy (0.6%), France (0%), and Sweden (0%)—the proportion remains exceedingly low.

An uptake [4] of robotic surgery within metabolic and bariatric surgery (MBS) appears justified, as the robotic platform offers potential solutions to technical difficulties such as limited intra-abdominal workspace, hepatomegaly, abundant visceral fat, and a thick abdominal wall [5, 6]. Moreover, in the absence of a standardized operative protocol, certain technical steps—such as stapling—are still, in some instances, completed laparoscopically.

The Italian Working Group on Robotic Bariatric Surgery analyzed national registry data (2014–2024), showing a steady rise in robotic bariatric procedures in Italy from 0.2% to 3.7% of all case, being robotic sleeve gastrectomy the most performed procedure in 2024 [7].

To advance beyond the current laparoscopic standard, a fully robotic sleeve gastrectomy (RSG)—in which *all* operative steps are performed with robotic instrumentation—should be adopted and standardized. At our institution, after an initial hybrid approach in the earliest cases, we progressed rapidly during the learning phase to performing the operation entirely robotically.

In a previous matched-pair analysis [8] comparing first 25 RSG cases with laparoscopic sleeve gastrectomy, similar perioperative outcomes were reported, with longer total operative time for RSG primarily due to robotic docking.

Recent studies [9, 10] on robotic sleeve gastrectomy (RSG) have shown that, despite higher costs and initially longer operative times, the learning curve leads to shorter procedures, lower conversion rates, and fewer complications compared to laparoscopy. Overall, RSG appears safe and effective, offering perioperative advantages once proficiency is achieved. With our work, we aim to contribute to this growing body of literature by focusing on the learning curve in robotic sleeve gastrectomy.

Aim of the present study was to analyse outcomes in terms of operative time, complications and cost of RSG during our learning curve.

## Materials and Methods

### Study Design and Patient Selection

We conducted a retrospective analysis of the first 53 consecutive patients who underwent totally robotic sleeve gastrectomy at our institution between April 2023 and July 2025. All procedures were performed by the same surgeon using a standardized robotic technique. There was no specific selection process for patients undergoing robotic sleeve gastrectomy; cases were included consecutively without additional criteria. At our center, the majority of bariatric procedures (> 200 annually) are currently performed laparoscopically, which remains the standard approach for the surgical team. Demographic and perioperative data were collected from electronic medical records, including age, BMI, docking time (time from patient positioning to console start), console time (time at the robotic console), total operative time (skin incision to closure), and length of stay. Categorical variables included sex, presence of complications, ICU admission, readmission, and postoperative medication use. Complications were defined as any deviation from the normal postoperative course requiring intervention. In the absence of a laparoscopic control group, outcomes were compared with the globally accepted 30-day benchmarks for the laparoscopic and robotic approach [11, 12]. Data collection was approved by the Institutional Review Board (approval record no. 9/24, June 12, 2024).

### Robotic Surgical Technique

All procedures were performed using the da Vinci Xi robotic platform with the patient in the supine position and legs apart, and the first assistant standing between the legs. Pneumoperitoneum was established via a Veress needle at Palmer’s point, followed by insertion of an 8-mm optical trocar on the midline, 15–20 cm from the xiphoid process, using an open technique. Three additional 8-mm robotic trocars were placed along the same horizontal line with an 8-cm distance between them, and a 12-mm accessory laparoscopic port was positioned in the left flank. The robot was docked from the patient’s left side.

The robotic arms were equipped as follows: Arm 1 with Cadiere or Pro-Grasp forceps (used as liver retractor and for traction), Arm 2 with fenestrated bipolar forceps (later replaced by the robotic stapler), Arm 3 with a 30° robotic camera, and Arm 4 with a Vessel Sealer for energy dissection.

The procedure began with entry into the omental bursa and division of the greater omentum along the greater curvature, starting 4 cm from the pylorus, using the vessel sealer. After complete mobilization of the gastric fundus and visualization of the left crus, a 36-Fr bougie was introduced along the lesser curvature to calibrate the sleeve. The robotic stapler was then mounted on Arm 2, and vertical gastrectomy was performed along the bougie.

The staple line was inspected for bleeding, and metallic clips were applied if necessary. A methylene blue leak test was routinely performed. The specimen was extracted through the accessory port, and a drain was placed along the staple line.

For the first 28 cases, green cartridges were used for the antrum and blue cartridges for the body and fundus. From case 29 onward, only blue cartridges were used for the entire staple line.

### Learning Curve Analysis

The learning curve was assessed using Cumulative Sum (CUSUM) analysis for docking time, console time, and total operative time [13]. For each metric, individual case values were subtracted from the overall mean and cumulatively summed. The resulting CUSUM plots were fitted with a third-order polynomial to identify inflection points corresponding to phase transitions. Based on these change-points, two phases were defined for each metric: Phase 1 (initial learning) and Phase 2 (proficiency).

### Cost Analysis

Direct instrument and stapling costs were calculated for the Intuitive platform configuration and compared with hypothetical costs using Ethicon™ and Medtronic™ energy and stapling devices. The mean number of cartridges was computed using the count of robotic cartridges as a reference.

### Statistical Analysis

Normality of continuous variables within each phase was tested using the Shapiro–Wilk test. Since at least one phase per metric deviated from normality, Mann–Whitney U tests (Wilcoxon rank-sum) were applied to compare Phase 1 and Phase 2 for operative time, docking time, and console time. Effect sizes were reported as rank-biserial correlation (r). A *p*-value < 0.05 was considered statistically significant. Descriptive statistics were expressed as mean ± standard deviation (SD) or median [interquartile range] as appropriate. Fisher’s exact test was used to compare rates.

## Results

A total of 53 patients underwent totally robotic sleeve gastrectomy. The mean age was 38.1 ± 12.6 years (range 19–63), and the majority were female (71.7%, *n* = 38). The mean BMI was 41.6 ± 6.3 kg/m^2^ (range 34.1–69). Mean docking time was 17.8 ± 9.4 min, console time 51.5 ± 17.0 min, and total operative time 69.3 ± 18.1 min. The mean length of stay was 3.6 ± 1.1 days (range 2–10) (Table 1).

**Table 1:** Demographic continuous variables

Variable	N	Mean	SD	Min	Max
Age (years)	53	38.09	12.64	19	63
BMI (kg/m^2^)	53	41.59	6.26	34	69
Docking time (min)	53	17.81	9.40	10	47
Console time (min)	53	51.51	16.97	27	122
Operative time (min)	53	69.32	18.13	42	135
Length of stay (days)	53	3.55	1.10	2	10

### Postoperative Outcomes

Overall, 4 patients (7.5%) experienced at least one postoperative complication, while the procedures were uneventful in 92.5%. The most frequent adverse event was staple-line bleeding (*n* = 3), followed by one leak. No stricture or mortality were observed, and no reintervention was needed. Three events occurred in the first 28 patients (10.7%) compared with none in the subsequent 25 patients (0%) (p ≈ 0.24).ICU admission occurred in 1 patient (1.9%), and readmission in 1 patient (1.9%). Antiemetics were administered in 18.9% of cases, and postoperative analgesics in 32.1% (Table 2).

**Table 2:** Demographic categorical variables

Variable	Category	N	%
Sex	Female	38	71.7
	Male	15	28.3
Complications	Yes	4	7.5
	No	49	92.5
ICU admission	Yes	1	1.9
	No	52	98.1
Readmission	Yes	1	1.9
	No	52	98.1
Antiemetics postop	Yes	10	18.9
	No	43	81.1
Analgesics postop	Yes	17	32.1
	No	36	67.9

### Comparison to Global Benchmarks

Operative performance met almost all internationally accepted 30‑day laparoscopic and robotic benchmarks, except for postoperative bleeding (Table 3).

**Table 3:** Comparison – Robotic sleeve gastrectomy vs laparoscopic and robotic benchmark (30 days)

Indicator (30 days)	Our data (*n* = 53)	LSG Benchmark	RSG Benchmark
Conversion to open	0%	0%	0%
Operation duration (mean)	69.3 ± 18.1 min	≤ 90 min	≤ 90 min
Hospital stay, median	3 days	≤ 3 days	≤ 2 days
Any complication until 30 days	7.5%	≤ 8%	≤ 9.1%
Overall readmission until 30 days	1.9%	< 4%	1.8%
Staple line leak	1.9%	0.2%	0.4%
Stenosis	0%	0%	0%
Post-op bleeding	5.7%	≤ 1.7%	0.4%
Bowel obstruction	0%	0%	0%
Wound infection	0%	0%	0%
Mortality	0%	0%	0%
Reoperation	0%	≤ 2%	0.8%

### Learning Curve Analysis (CUSUM)

CUSUM analysis was performed for docking time, console time, and overall operative time. Each metric showed a distinct inflection point, indicating the transition from the initial learning phase to a more stable performance phase:

-*Docking time:* learning point at case 18; mean decreased from 26.9 min (Phase 1, cases 1–18) to 13.1 min (Phase 2, cases 19–53), a 51.1% reduction.

-*Console time:* learning point at case 23; mean decreased from 57.6 min (Phase 1, cases 1–23) to 46.9 min (Phase 2, cases 24–53), an 18.6% reduction.

-*Operative time:* learning point at case 22; mean decreased from 79.3 min (Phase 1, cases 1–22) to 62.2 min (Phase 2, cases 23–53), a 21.5% reduction.

### Statistical Comparison Between Phases (Mann–Whitney U)

Because at least one phase for each metric deviated from normality at Shapiro–Wilk testing, we compared Phase I vs Phase II using two-sided Mann–Whitney U (Wilcoxon rank–sum). Effect size is reported as rank-biserial r (negative values indicate lower values in Phase II) (Table 4).

**Table 4:** Comparison between phase I and II

Metric	Phase 1 n	Phase 2 n	U statistic	*p*-value	Rank-biserial r	Interpretation
Operative time (min)	22	31	561.0	0.000073	− 0.65	Significant (p < 0.05); lower in Phase II
Docking time (min)	18	35	567.5	0.000002	− 0.80	Significant (p < 0.05); lower in Phase II
Console time (min)	23	30	453.5	0.0523	− 0.31	Not significant (p ≥ 0.05)

### Cost Analysis

Using Intuitive/Da Vinci instruments, the average cost per procedure was €4,535.83, including one single-use vessel sealer (€941), one Cadiere forceps amortized over 18 uses (€155.94), one bipolar forceps amortized over 14 uses (€294.50), an average of 6.89 60-mm stapler cartridges (€351 each; €2,418.39), and one SureForm 60-mm stapler (€726). For 53 procedures, the total expenditure was €240,399.23.

Assuming the use of Ethicon components, the estimated average cost per procedure was €2,517.35, including one ECHELON Powered 60-mm stapler (€360), one Harmonic ACE (€676), and an average of 6.89 ECHELON GST Reload cartridges (€215 each; €1,240.20). For 53 procedures, the total cost would be approximately € 133,419.55.

Using Medtronic components, the estimated cost per procedure was €, including one Ligasure device (€510), one Signia stapler handle (€46,67), one Signia linear adapter (€46), one shell (€218) and an average of 6.89 TriStaple cartridges (€154 each; €1,060.06) (Table 5).

**Table 5:** Cost Comparison per Sleeve Gastrectomy and Savings

Configuration	Main Components Included	Avg. Cost per Case (€)	Total for 53 Cases (€)	Savings vs Intuitive (per case)	Savings vs Intuitive (total)
Intuitive/Da Vinci	Vessel sealer, Cadiere, Bipolar, SureForm cartridges, SureForm stapler	4,535.83	240,399.23	–	–
Ethicon	ECHELON stapler, Harmonic ACE, ECHELON GST Reload cartridges	2,444.20	129,543.00	2,091.63	110,856.23
Medtronic	Ligasure, Signia handle, Signia shell, TriStaple cartridges	1,880.73	99,678.69	2,655.10	140,720.54

## Discussion

Over the past several years, our research group has cultivated a sustained interest in understanding how surgical proficiency develops in bariatric procedures. A previous study analysed the first 100 laparoscopic sleeve gastrectomies performed by a newly trained surgeon, assessing perioperative outcomes against global benchmark standards. CUSUM analysis showed that most benchmarks were achieved after 50 cases, with operative time and hospital stay plateauing after slightly more procedures. The findings confirm that LSG is feasible and safe in structured training programmes under senior supervision [14].

A systematic review by Hirri et al [15], including 11 studies and a total of 1,237 patients undergoing robotic bariatric surgery, identified a learning curve ranging from 11 to 84 cases, with a progressive and statistically significant reduction in operative time in most series. Only a minority of the studies reported a relevant decrease in major morbidity after the learning curve was completed. The overall complication rate was approximately 7–8%, with no mortality and a very low incidence of leaks—parameters that meet the recently proposed international benchmark criteria for robotic metabolic surgery. These findings suggest that, in settings with standardised technical protocols and dedicated teams, achieving benchmark-level quality is feasible even in the early phases of the learning process.

An 8-year MBSAQIP analysis (2015–2022) of 823,902 primary bariatric cases found that robotic sleeve gastrectomy (R-SG) had higher 30-day morbidity, leak, and bleeding rates than laparoscopic SG. However, recent outcomes for robotic cases have moved closer to laparoscopic results, though some complication rates remain higher [16].

Evidence from a prospective cohort study comparing robotic-assisted and laparoscopic sleeve gastrectomy demonstrated comparable operative times and complication rates, but a significantly shorter length of stay for the robotic group (1.3 vs. 1.9 days). Consumable costs were unexpectedly lower for robotic procedures (€2,310 vs. €2,665). The learning curve for robotic sleeve gastrectomy was estimated at 26 cases, with the stapling phase being the main driver. These findings suggest that, when using advanced robotic stapling and energy devices, robotic sleeve gastrectomy can be safe, cost-effective, and associated with enhanced recovery [10].

Our analysis of the first 53 totally RSGs cases demonstrated that, even during the initial implementation phase, perioperative morbidity remained within an acceptable range and aligned with internationally recognized quality standards for laparoscopy, with the sole exception of postoperative bleeding. The observed bleeding rate (5.7%) was higher than benchmark thresholds and occurred in the early phase of the series. Although this difference did not reach statistical significance, it raised the possibility of staple height–tissue mismatch, particularly in thicker gastric tissue at the antrum. The subsequent shift to blue loads was therefore a pragmatic, safety‑oriented adjustment rather than a conclusion grounded in definitive evidence.

Importantly, also our outcomes in terms of operative time remained within these benchmark thresholds throughout the entire learning curve. Learning curve analysis (CUSUM) indicated proficiency for total operative time was reached after approximately 20–22 cases, with docking time improving earliest (case 18) and representing the principal factor in longer initial procedures. The additional operative duration related to docking averaged about 10 min compared to laparoscopy—an increase that is unlikely to be clinically significant from an anesthesiological or surgical risk perspective. Console time reductions were more gradual, suggesting a stepwise acquisition of platform-specific technical skills.

From an economic perspective, while RSG currently entails higher direct instrument and stapling costs than laparoscopic sleeve gastrectomy, the expansion of the competitive robotic market is expected to reduce expenditure over time, potentially achieving cost parity. Given the procedural safety and the potential ergonomic and technical advantages of the robotic platform, especially in complex or revisional settings, the anticipated cost evolution further supports the integration of RSG into routine bariatric practice.

Although our initial robotic experience was limited to a few cases per month, in the last six months our activity has increased to approximately two procedures per week. These data may encourage institutions that cannot start with high robotic volumes to progress gradually while maintaining safety and quality standards.

Indeed, docking times were initially prolonged, reflecting the start-up phase of our robotic program and variability in team composition and training. This highlights that the learning curve can be safely overcome after about 20 cases even when starting from zero, encouraging other centers beginning their robotic experience.

Our current direction is the continuation of our ethically approved research project aimed at developing a randomized trial comparing robotic versus laparoscopic sleeve gastrectomy. This study will be essential to determine whether the two approaches are truly equivalent in terms of safety and efficacy.

### Strengths and Limitations

The strength of this study is to underline that the initial obstacle of the robotic learning curve should not discourage its adoption: early minor complications are expected with a new approach, but as long as international safety standards are maintained, once proficiency is achieved outcomes can match those of laparoscopy. By applying a structured CUSUM analysis to discrete procedural metrics—docking time, console time, and total operative time—this work provides objective thresholds for the transition from the initial learning phase to procedural proficiency. Limitations include the retrospective design, the relatively small sample size inherent to early implementation series, and the absence of long-term follow-up for weight-loss or metabolic outcomes. As this was a retrospective study, we were unable to precisely account for all disposable materials; consequently, the cost analysis presented provides only a partial estimate.

## Conclusion

**Fig. 1:**
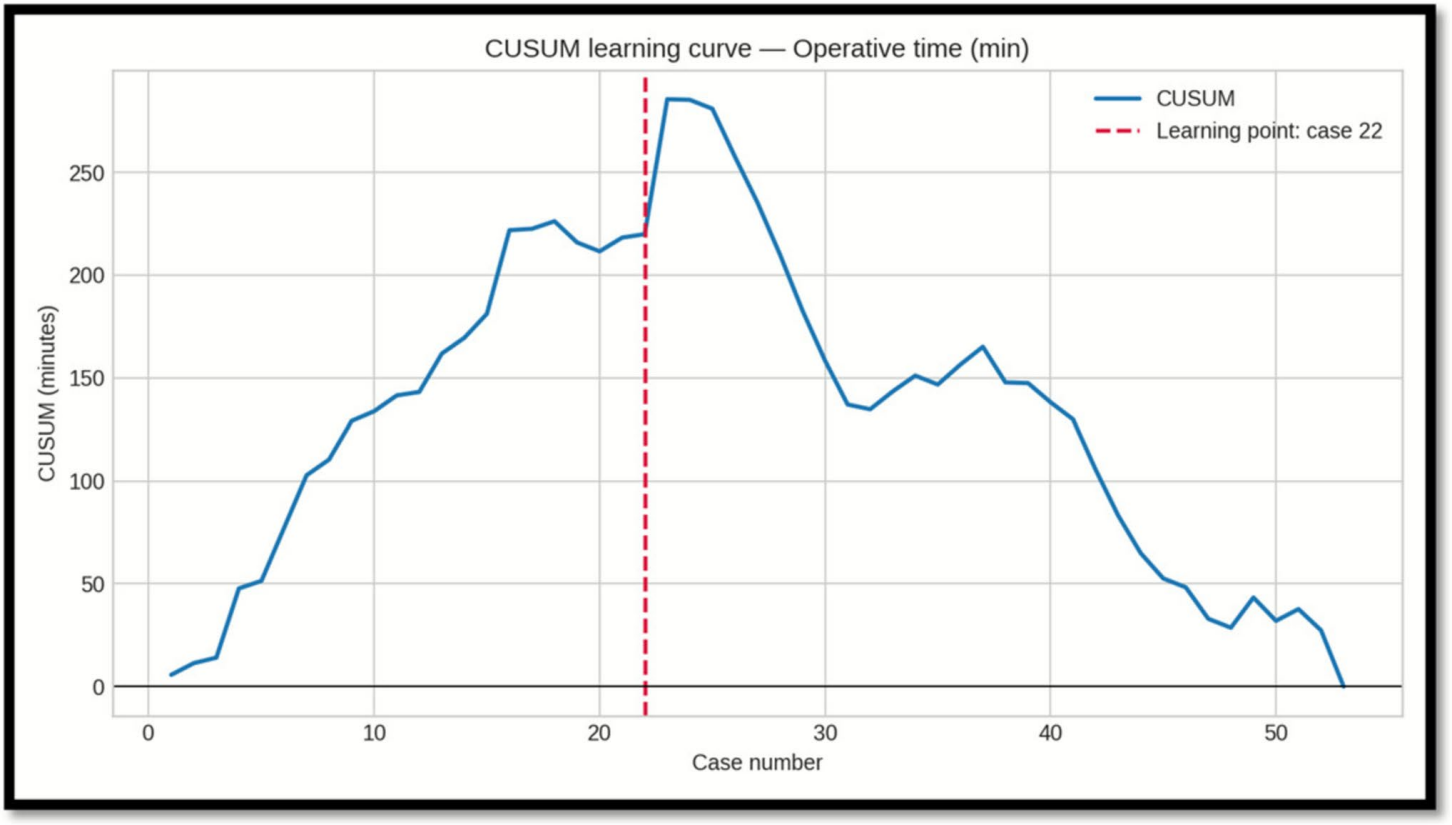
CUSUM curve for operative time

**Fig. 2:**
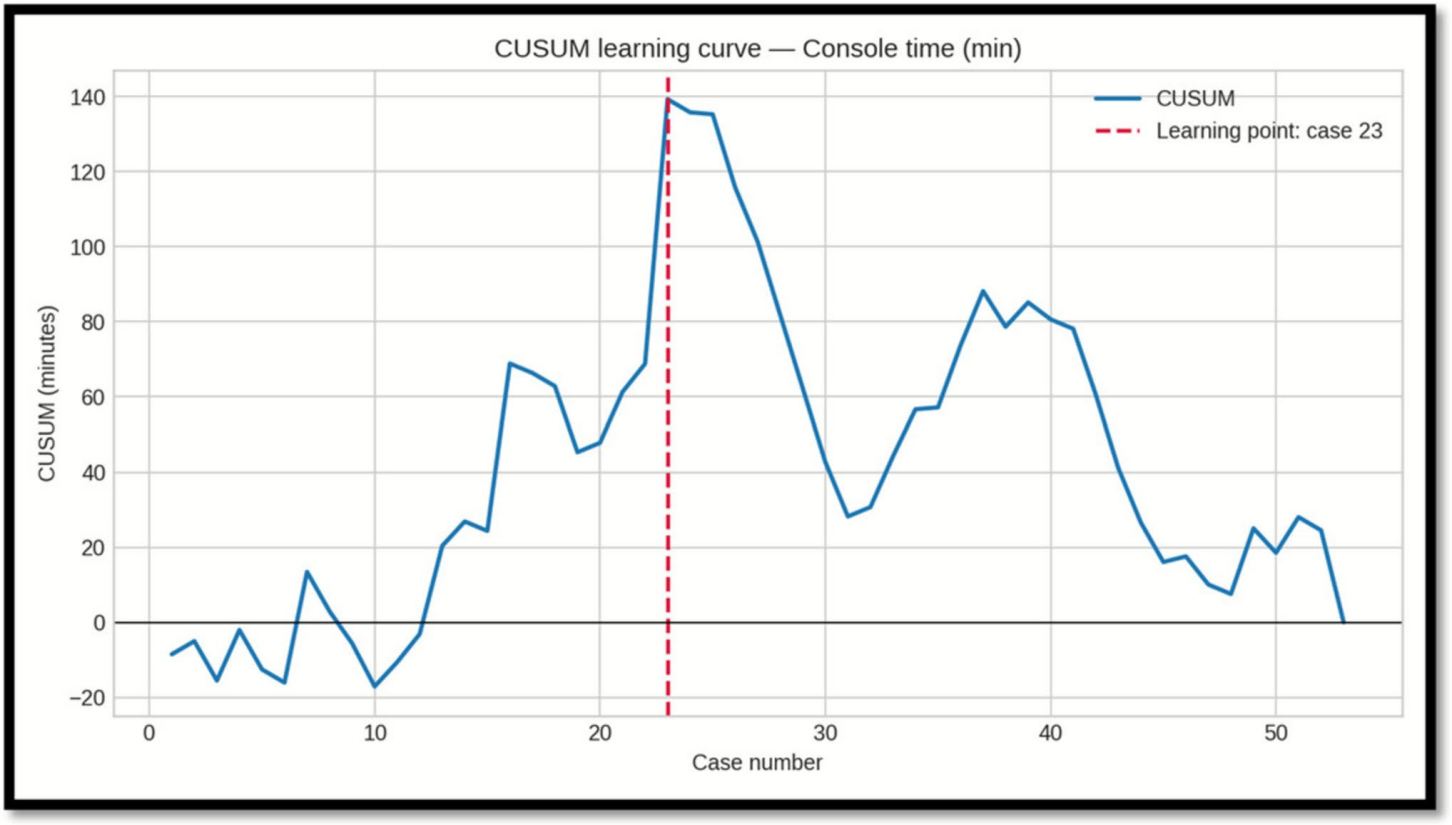
CUSUM curve for console time

**Fig. 3:**
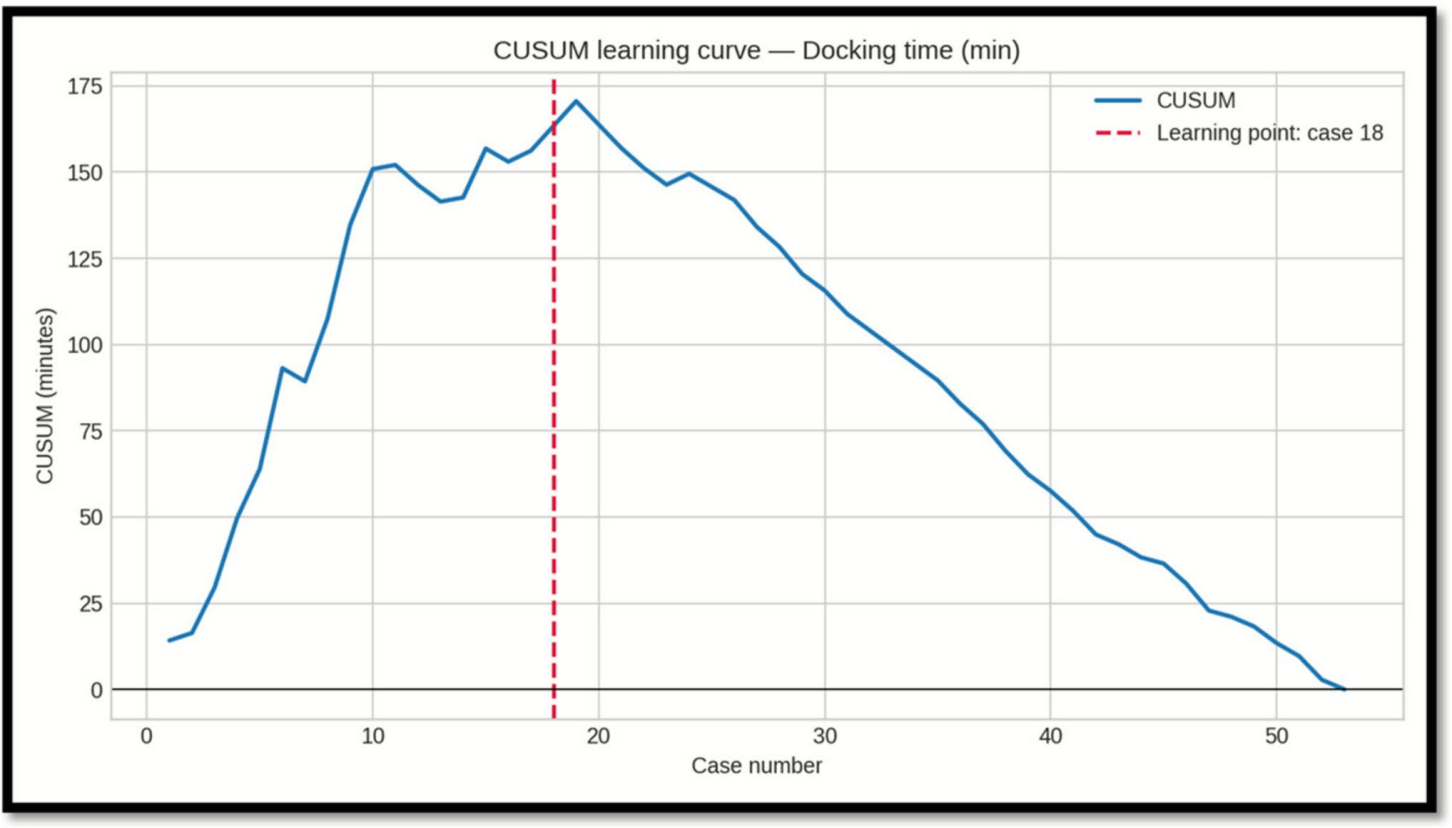
CUSUM curve for docking time

Totally robotic sleeve gastrectomy can be introduced safely within an experienced bariatric program, achieving perioperative outcomes that are acceptable when comparted to international benchmark standards for laparoscopy even during the learning phase. Proficiency in operative efficiency—particularly through optimisation of the docking process—was reached after approximately 20 cases, while complication rates remained low and no mortality occurred. These results, coupled with potential strategies for cost containment, support the integration of RSG as a viable alternative to conventional laparoscopy, warranting further multicentre studies to confirm external validity and assess long-term clinical benefits (Figs. 1, 2 and 3).

## Data Availability

No datasets were generated or analysed during the current study.
